# Native and Prosthetic Simultaneously Double Valve Infective Endocarditis with *Enterococcus faecalis*—Case-Based Review

**DOI:** 10.3390/jpm13020300

**Published:** 2023-02-08

**Authors:** Raluca Ecaterina Haliga, Victorita Sorodoc, Bianca Codrina Morarasu, Adorata Elena Coman, Alexandr Ceasovschih, Oana Sirbu, Catalina Lionte, Cristina Bologa, Alexandra Stoica, Mihai Constantin, Gabriela Puha, Ovidiu Rusalim Petris, Minerva Codruta Badescu, Daniela Crisu, Andreea Nicoleta Catana, Ioana Bianca Haliga, Laurentiu Sorodoc

**Affiliations:** 1 Faculty of Medicine, Department of Internal Medicine, “Grigore T. Popa” University of Medicine and Pharmacy, 700115 Iași, Romania; 2 2nd Internal Medicine Clinic, “St. Spiridon” Emergency Clinical County Hospital, 700111 Iași, Romania; 3 Preventive Medicine and Interdisciplinary Team Department, “Grigore T. Popa” University of Medicine and Pharmacy, 700115 Iasi, Romania; 4 Nursing Department, “Grigore T. Popa” University of Medicine and Pharmacy, 700115 Iasi, Romania; 5 3rd Internal Medicine Clinic, “St. Spiridon” Emergency Clinical County Hospital, 700111 Iași, Romania; 6 Department of Cardiology, “St. Spiridon” Emergency Clinical County Hospital, 700111 Iași, Romania; 7 Department of Infectious Diseases, “St. Spiridon” Emergency Clinical County Hospital, 700111 Iași, Romania

**Keywords:** double valve infective endocarditis, mitral prosthetic valve, aortic native valve, *Enterococcus faecalis*

## Abstract

Infective endocarditis is a severe infective heart disease, commonly involving native or prosthetic valves. It frequently presents with univalvular involvement and simultaneous double valve or multivalvular involvement is rarely described. The third leading cause of infective endocarditis worldwide is *Enterococcus faecalis*, which is associated with high mortality rates despite important advances in antimicrobial therapy. It develops secondary to enterococcal bacteremia, with its origin from the gastrointestinal or genitourinary tract and predominantly affecting the elderly population with multiple comorbidities. Clinical presentation is usually less typical, and the treatment is challenging. It can be marked by antibiotic resistance, side effects, and subsequent complications. Surgical treatment can be considered if deemed appropriate. To the best of our knowledge, we present the first case-based narrative review of *Enterococcus faecalis* double valve endocarditis involving both the aortic native and prosthetic mitral valve, highlighting the clinical characteristics, treatment, and complications of this condition.

## 1. Introduction

Infective endocarditis (IE) is a potentially fatal condition affecting the endocardial surface. It generally involves a native or prosthetic heart valve or a cardiac device. The epidemiology has greatly changed over the past few years, probably due to the proportion of invasive interventions, longer life expectancy, and associated comorbidities. Currently, the incidence of IE varies from three to seven cases per 100,000 population yearly. It seems to commonly affect male individuals, with an average age of 67 years old, but younger groups with risk factors, such as intravenous (IV) drug use, are at risk. Despite the wide availability of antimicrobial therapy, the mortality rate is still high, at 14–46% at one year post-infection [1,2].

Rheumatic heart disease remains a major risk factor in low-income countries. Its prevalence has declined in developed countries nowadays and is being replaced by risk factors such as structural heart disease, degenerative or congenital conditions, prosthetic valve or cardiac device use, a history of IE, immunosuppression, intravenous drug use, chronic hemodialysis, or undergoing recent invasive procedures [1,3]. Healthcare-associated endocarditis is now responsible for around 25 to 30% of IE cases. Surgical interventions, such as the correction of congenital heart disease and the insertion of prosthetic valves or cardiac devices, including implantable cardioverters or pacemakers, have led to major changes in this condition. The involvement of native valves usually requires a damaged structure, and it commonly affects the mitral valve [4,5,6]. Rare presentations may include a concomitant infection of two valves [3], but studies and case reports have not described the simultaneous involvement of native and prosthetic valves.

The disease has a wide spectrum of clinical presentation. It can present with specific signs, such as fever, new heart murmurs or increasing intensity of previous murmurs, splenomegaly, or anemia. A less typical clinical presentation is increasingly prevalent among elderly patients with important comorbidities [7]. IE should be considered in every patient with pyrexia or sepsis of an unknown source and in patients with an unexplained stroke, systemic embolism, or heart failure (HF). General symptoms such as malaise, chills, low-grade fever, and joint pain may be the only complaint. The classical microembolic and immunologic features are less common [8].

To the best of our knowledge, we present the first case-based narrative review of *Enterococcus faecalis* double valve endocarditis involving both the stenotic aortic native valve and the prosthetic mitral valve and highlighting the etiology, clinical presentation, treatment, and complications of this condition.

## 2. Case Presentation

We present the case of a 73-year-old female referred to our Emergency Department (ED) with hypotension, vertigo, and fatigue. She has a complex past medical history of permanent atrial fibrillation, previous mitral valve replacement with a mechanical valve implanted 10 years ago, chronic microvascular cerebral ischemic disease, anemia, and hypothyroidism. Her social history is unremarkable, with no smoking or alcohol consumption. Chronic medication included Acenocumarol, without regular INR monitoring, Carvedilol, Candesartan, and Levothyroxine. The patient was previously admitted to the regional Psychiatry Hospital with newly diagnosed mixed vascular-Alzheimer dementia. While there, she developed a low blood pressure (BP) of 70/40 mmHg and was immediately referred to our hospital.

Upon the patient’s arrival in ED, she was hemodynamically unstable with hypotension (BP 85/55 mmHg), hence the immediate correction with intravenous (IV) macromolecular solutions was started. Concomitant initial investigations were performed (chest and abdominal x-ray, abdominal ultrasound) without significant findings. Consequently, the patient was admitted to our internal medicine clinic with a negative RT-PCR for SARS-CoV2.

On initial assessment, the patient was generally unwell, with pale skin and mucous membranes, irregular heart sounds, a systolic murmur in the aortic foci, a metallic prosthetic click in the mitral area, and chronic skin venous changes in the lower limbs. Her heart rate was 88 with normal oxygen saturation and temperature. Electrocardiogram (ECG) showed atrial fibrillation at a ventricular rate of 80 beats per minute without ischemic changes. Blood tests revealed moderate normochromic normocytic anemia (Hb 8.1 g/dl), mild thrombocytopenia (121,000/µL), subtherapeutic INR (1.21), mild inflammatory syndrome (CRP 2.99 mg/dl), and a high level of N-terminal pro-brain natriuretic peptide (NTproBNP 8586 pg/mL). Midstream urine analysis was consistent with a urinary tract infection, with subsequent urinary cultures testing positive for *Enterococcus faecalis*.

**Figure 1:**
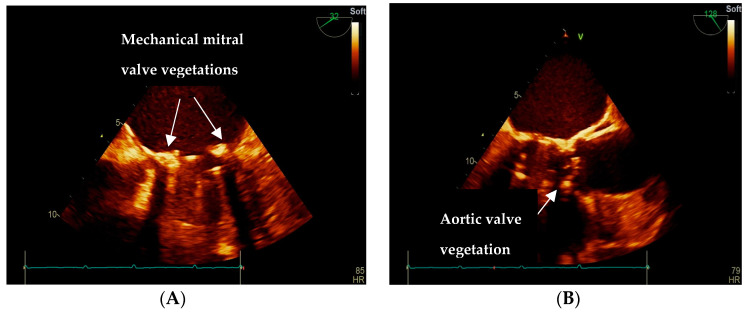
Transesophageal echocardiography. (**A**) Anterior and posterior mitral mechanical valve insertion vegetations. (**B**) Vegetation of the aortic non-coronary cusp (from Cardiology Clinic, “St. Spiridon” Emergency Clinical County Hospital, Iasi, Romania).

Immediate treatment consisted of IV fluids, broad-spectrum antibiotics (Ceftriaxone), and anticoagulation with low molecular weight heparin (LMWH). After appropriate IV hydration and BP correction, we initiated beta-blocker therapy for heart failure (HF) and rate control in atrial fibrillation and switched the LMWH to Acenocoumarol, with INR monitoring. Within the third day of admission, the patient developed pyrexia (38.3° Celsius body temperature), vertigo, and fatigue. In this context, two consecutive blood cultures were collected, which came back positive for *Enterococcus faecalis.* A transthoracic echocardiography (TTE) was performed, revealing global dilation of cardiac cavities, the normal systolic function of the left ventricle with a preserved ejection fraction of 50%, moderate degenerative aortic stenosis, a normofunctional mitral mechanical valve, 3rd-degree tricuspid regurgitation, and severe pulmonary hypertension. To rule out IE, a transesophageal echocardiography (TOE) was performed and detected two vegetations of 5/4 mm and 6/4 mm, located at the anterior and posterior mitral mechanical valve insertion, respectively, and another of 5/5 mm of the aortic non-coronary cusp (Figure 1A,B).

According to Duke’s criteria, the patient was diagnosed with double valve IE, affecting the aortic stenotic native valve and mitral mechanical valve. Following blood culture results and sensitivity, IE guidelines, and infectious disease (ID) specialist advice, we switched antibiotics to Ampicillin and Gentamicin. We noted a good therapeutic response for the following two weeks until the inflammatory syndrome peaked again, and renal function deteriorated. Antimicrobial agents were switched to an association of imipenem/cilastatin and vancomycin in doses adjusted to renal function. We considered the necessary continuation of vitamin K antagonist (VKA) anticoagulation, however, there were labile INR values and limited hemorrhagic complications (nose bleedings) requiring local hemostatic treatment.

After the following 14 days, the anemic syndrome and thrombocytopenia worsened (Hb 5.6 g/dL, platelet count 42,000/µL). We considered the blood cytopenia secondary to imipenem and vancomycin treatment. We corrected it with the administration of red blood cell concentrates, repeated standard platelet concentrates, and an antibiotic switch to teicoplanin for another 14 days.

TOE was performed after 6 weeks of hospitalization and treatment, showing the reduced size of aortic non-coronary cusp vegetation to 3mm, with a resolution of mitral mechanical valve insertion vegetations. The clinical evolution was favorable and supported by blood tests and imagistic investigations. The patient was discharged in stable condition with outpatient multidisciplinary regular follow-ups.

## 3. Discussion

### 3.1. Etiology of Double Valve Infective Endocarditis (DVIE)

*Streptococcus viridans* and *Staphylococcus aureus* collectively account for the etiology of 80% of native valves IE, while 10% are considered culture-negative [3]. Fewer studies analyzed patients with DVIE [9,10], but in a recent one including 1340 patients with acute left-sided native valve IE, 19% of patients had bivalvular involvement. The etiology was predominantly represented by *Streptococcus* spp. or *Enterococcus* spp. [11]. In another retrospective analysis of the Spanish Registry for IE, including 4064 definite cases of valvular IE during twelve years, 14.2% of patients had multivalvular infective endocarditis (MIE). These involved mitral and aortic native valves with no prosthetic valve cases. The most common etiologies were *Streptococcus viridans* and *Staphylococcus aureus* [12].

### 3.2. Site of Infection

The mitral valve is the most common site of infection. The involvement of two or more cardiac valves is less frequently described [3], and it may predominantly affect the mitral and aortic valves [5]. When DVIE occurs, it results in more severe and extensive cardiac lesions, extracardiac complications [9], and a greater risk of mortality [5]. Prosthetic valve endocarditis (PVE) is the most severe form of IE, accounting for approximately 20% of cases [13], with bioprosthetic valves at higher risk [14]. It seems that the type of intervention does not influence the incidence as transcatheter aortic valve implantations (TAVI) and surgical implantations pose similar risks of IE. It can occur early, within a year after the implantation, or later, when it carries comparable risks with native valve endocarditis [15].

### 3.3. IE with E. faecalis and Sources of Infection

*Enterococcus faecalis* (*E. faecalis*) is the third leading cause of IE worldwide, accounting for 5–15% of all IE cases [16]. It is a severe infection with high mortality rates (20–40%), despite the important advances made in antimicrobial therapy [13,17,18]. *E. faecalis* IE (EFIE) has been mainly described in an elderly population with cardiac or other comorbidities [7]. It predominantly involves the left heart and poses a higher risk of periannular abscesses and prosthetic valve involvement. Approximately 40% of cases require surgical intervention, which is in contrast to other types of IE [19,20,21]. Enterococcal bacteremia usually originates from the gastrointestinal or genitourinary tract [22], secondary to urinary tract infections and diagnostic or therapeutic procedures (urinary catheter insertion, cystoscopy, or transurethral resection of the bladder or prostate) with the same risk profile for the gastrointestinal tract [16,18]. Our patient was diagnosed with an *E. faecalis* urinary tract infection. We considered this site of infection as the source of bacteremia and subsequent IE.

### 3.4. Positive Diagnosis of EFIE

Approximately 75–90% of cases of IE present with fever and cardiac murmurs. The classic microembolic and immunologic features, such as Osler nodes, Roth spots, Janeway lesions, or splinter hemorrhages, are rarely described and only present in around 5–10% of cases. Hematogenous seeding can involve the spleen, kidneys, musculoskeletal system, meninges, or skin. In approximately 50% of cases, an embolic phenomenon leading to end-organ ischemia can develop. This is frequently related to *Staphylococcus aureus*-associated IE, with the possible formation of large (more than 1 cm) and mobile vegetations involving the anterior leaflet of the mitral valve [1].

In contrast, EFIE poses diagnostic challenges due to its subacute course and nonspecific symptoms of fever, malaise, and generalized aches. The septic screen might be performed multiple times as it is difficult to distinguish from other infectious diseases [23].

The gold standard diagnosis is based on the modified Duke criteria, a sensitive and specific tool for IE, and major and minor criteria [3]. Major clinical criteria comprise positive blood cultures with typical microorganisms, such as Staphylococcus aureus, streptococcus viridans, HACEK group, and community-acquired enterococci; microorganisms may determine IE with specific time intervals and the number of blood cultures. For Coxiella Burnetii etiology, a single positive blood culture or IgG antibody titre > 1:800 confirms the diagnosis. Echocardiography is a major criterion when it identifies either vegetation, an abscess, or a new partial prosthetic valve dehiscence. Minor criteria include a predisposing heart condition or IV drug use, temperature ≥ 38 °C, vascular or immunologic phenomena, and positive blood culture without characteristics of major criteria [8]. The modified Duke criteria are, however, less sensitive when blood cultures are negative, or the infection involves prosthetic valves, cardiac devices, or the right heart [1]. The clinical presentation in our patient was unsuggestive for IE, as the patient presented with vertigo, fatigue, and hypotension, considered in the context of urinary sepsis. However, our case associated multiple risk factors for IE, out of which the presence of a mechanical valve is a key one.

Blood cultures are the most important biological components of major criteria, establishing a causative microorganism and guiding antibiotic therapy. Around 90% of native valve endocarditis (NVE) have positive cultures. Blood cultures can be taken at any moment due to continuous bacteremia, and the aseptic technique is particularly important as it can distinguish skin contaminant microorganisms. Another 10% of cases are culture-negative in the context of recent antibiotic treatment, difficult-to-grow microorganisms, or non-bacterial thrombotic endocarditis. Repeat blood cultures and other types of laboratory techniques may be used to identify fastidious organisms [24]. Recent studies showed that a quarter of the patients diagnosed with *E. faecalis* bacteremia have IE [25]. On the other side, a short time to blood culture positivity should raise suspicions of a high bacterial blood concentration and be associated with an increased risk of IE [26]. This should prompt the clinician to perform echocardiography in all patients with *E. faecalis* bacteremia.

TTE plays a central role in the major criteria, and it should be performed on every patient with a strong clinical suspicion. TTE can identify new valvular regurgitation, vegetations, abscesses, or new dehiscence of prosthetic valves. It has a sensitivity of 75% and a specificity of 90%. Vegetations can be identified on the upstream surfaces of the heart valves, which may lead to systemic embolic complications [27]. If TTE is negative or non-diagnostic, especially in the presence of a prosthetic heart valve or an intracardiac device, TOE is recommended. The latter has a sensitivity of over 90% [1]. TOE is generally necessary in the case of prosthetic valves, as the sensitivity of TTE is reduced to 36–69% [28]. In our patient, the vegetations were identified after TOE was performed.

### 3.5. Antibiotic Treatment in IE with E. faecalis

Enterococci are naturally tolerant to a number of antimicrobial compounds. They adapted to antibiotic-induced killing, hence requiring prolonged administration (4 to 6 weeks) of bactericidal combinations with synergistic action. Treatment of EFIE is particularly troublesome and challenging, with possible complicated evolutions and high mortality rates [18]. It seems that the microorganisms have developed multiple genetic and phenotypic changes within the valvular micro-environment. When compared to excised valvular specimens by sequencing methods, *E. faecalis* demonstrated different strains with a high diversity of virulence genes including deletions of ebpA, ebpB, ebpC, and srtC. This could explain the higher duration of infection and replacement rates or the ability to escape the host immune response [29].

The American Heart Association (AHA) [30] and European Society of Cardiology (ESC) guidelines are currently recommending a treatment combination of a cell wall synthesis inhibitor (β-lactam or vancomycin) with aminoglycosides (preferably gentamicin) [13]. In vitro studies showed bactericidal synergism between β-lactams and aminoglycosides and subsequently confirmed by clinical studies [31,32]. This led to an improvement in IE cure rates by up to 75% [7]. ESC guidelines recommend shortening the length of treatment with gentamicin from four to six weeks to two weeks [13,17]. The recommendation arose secondary to the increasing incidence of high-level aminoglycoside resistant (HLAR) EF and the risk of nephrotoxicity. The deterioration in renal function may be severe and partially reversible, especially due to the profile of patients with EFIE. Reducing the length of gentamicin treatment should decrease the adverse events while maintaining clinical outcomes and low relapsing rates. Based on the same rationale, different dosing intervals have been studied, and although there is a lack of randomized clinical data, once-daily administration is increasingly used [33,34,35].

Ampicillin plus ceftriaxone can be considered an alternative treatment option. The combination is relatively safe, without nephrotoxicity, and the main treatment for HLAR EFIE. The association inhibits penicillin-binding proteins 2 and 3, having a complementary effect and creating a bactericidal effect [23]. However, mortality rates continue to be high, as ceftriaxone, unlike other cephalosporins, has high biliary concentrations and promotes resistance and colonization with vancomycin resistant-enterococci—VRE and *Clostridium difficile* infections [7]. Antimicrobial treatment for PVE is similar to that of NVE, with the exception of PVE with *Staphylococcus aureus* [13].

Other treatment options have been studied in small cohorts. Teicoplanin showed better efficacy when compared to vancomycin, it allows a once-daily administration regime and may be used after 2 weeks of a standard combination antibiotic treatment [36]. Linezolid can be used in multidrug-resistant EFIE, especially in VRE faecalis strains, but conflicting data have been reported [37,38]. Other options, such as daptomycin [39] and amoxicillin/clavulanate plus cefditoren [40], could be used.

We initiated the antibiotic treatment according to blood culture sensitivity (*E. faecalis* sensitive to ampicillin, gentamicin, linezolid, vancomycin, teicoplanin, and tigecycline, intermediate sensitive to penicillin) and IE guidelines. The therapeutic combination of ampicillin and gentamicin was administered for two weeks. Follow-up blood tests showed alterations in renal function, considered secondary to aminoglycoside treatment, and persistent inflammatory syndrome, which led to an antibiotic switch in conjunction with the guidance of the ID specialist. A carbapenem (imipenem/cilastatin), in combination with vancomycin, was started and the doses were adjusted according to renal function. Vancomycin blood concentrations could not be monitored.

During the second course of antibiotics, INR levels increased, and thrombocytopenia worsened. We considered that both antibiotic drugs (imipenem/cilastatin and vancomycin) and simultaneous administration of anticoagulation contributed to hematological side effects. Out of the two antimicrobial agents, vancomycin has been reported to induce thrombocytopenia (vancomycin-induced thrombocytopenia—VIT) with a prevalence of 5.9 to 7.1%. The data is, however, limited, as the real incidence is still unknown due to the lack of large studies and high variability among existing ones [41]. Concomitant treatment with other agents may promote VIT, including imipenem [42]. The underlying mechanism seems to involve hapten-dependent antibody formation due to vancomycin adherence to platelet glycoproteins [43,44]. Other case reports of imipenem/cilastatin-induced thrombocytopenia have been reported [45,46], although it is considered a rare adverse event.

The general management includes discontinuation of the causative agent. We stopped the treatment with both antibiotic agents and switched to a new one (teicoplanin), together with the administration of red blood cell concentrates and repeated standard platelet concentrates. These therapeutic interventions corrected anemic syndrome and thrombocytopenia within seven days. Our patient’s renal function returned to baseline since we started vancomycin treatment, as impaired renal function may contribute to VIT and its slow resolution due to slow drug clearance. The literature describes other therapeutic measures, including prednisolone, intravenous immunoglobulins, rituximab, or plasma exchange [41].

### 3.6. Surgical Management

ESC Guidelines class I indications for the surgical management of native aortic and mitral valve IE include severe acute valvular regurgitation or obstruction, causing cardiogenic shock or refractory pulmonary edema, locally uncontrolled infection (fistula, abscess, false aneurysm), enlarging vegetation, as well as an infection caused by fungi or multiresistant organisms. Persistent vegetations of the aortic or mitral valve with a diameter of more than 10 mm, despite appropriate antibiotic therapy, are indications for surgery due to systemic embolic risks. Particular cases might be considered for early surgery. This can pose an increased risk of postoperative complications due to a lack of complete sterilization of the valve. This is less available for *Enterococcus*, particularly vancomycin-resistant and *Staphylococcus* endocarditis, due to a lack of correlation between the duration of pre-operative antibiotic treatment and the positivity of blood cultures [47].

The optimal therapeutic approach for PVE is still under debate. The guidelines mention that surgery for PVE follows the same principles indicated for NVE. In the Euro Heart Survey, surgery was performed in only 50% of patients with PVE, with similar rates being described for patients with NVE [48]. However, surgery was beneficial in the subgroup of patients with complicated evolution, including refractory congestive HF, severe prosthetic dysfunction, or enlarged vegetation [49]. Patients with uncomplicated non-staphylococcal and non-fungal late PVE can be approached by pharmacological management with antibiotic therapy [50]. However, these patients require close monitoring due to the risk of late complications [51].

Our patient did not present any of the systemic or local complications of IE. Significant therapeutic response by partial resolution of the IE vegetations has been observed following the antimicrobial treatment. Hence, surgical intervention was not indicated. The complications presented by the patient (worsening renal failure, anemia or thrombocytopenia, uncontrolled INR values, and bleedings) were consecutive to antibiotic and anticoagulation treatment.

### 3.7. Anticoagulant Treatment in Patients with IE

Current evidence does not support the initiation of anticoagulation or antiplatelet treatment as adjunctive therapy for IE [52]. Previous experimental and clinical studies showed a benefit from antiplatelet treatment by reducing complications, such as embolisms, the need for surgery, and mortality, but at the expense of increased bleeding risk [53,54,55]. On the contrary, cerebral embolism had a higher rate in patients treated with Aspirin compared to a placebo. Oral anticoagulation follows similar principles in IE, as it does not prevent the rate of cardioembolic stroke and increases the risk of bleeding [56].

Some authors consider that brain magnetic resonance imaging (MRI) should be performed irrespective of clinical status due to possible occult disease [57]. In native and metallic PVE, the continuation of anticoagulation was associated with decreased thromboembolic risk [58] and no increase in cerebral hemorrhagic complications [59]. A decision for discontinuation of anticoagulant therapy should be considered on an individual basis in patients with prior ischemic stroke, very large areas of infarction, and/or evidence of possible hemorrhagic transformation, coagulation disorders, or severe hypertension. Close monitoring of coagulation parameters is recommended. For patients with a past history or high risk of cerebral emboli, a lower INR target (i.e., 2.5–3) may be established.

In the case of previously anticoagulated patients, especially those with PVE, switching to therapy with unfractionated heparin under close monitoring of activated partial thromboplastin time for approximately two weeks is recommended [56]. LMWH can also be used, with the advantage of fewer side effects and no need for coagulation parameters monitoring. Direct oral anticoagulants are contraindicated in prosthetic heart valves due to inferiority compared to VKA. There have been fewer studies in IE cohorts, although Dabigatran may attenuate the activation of coagulation in *Staphylococcus aureus* bacteremia [60].

In our patient, oral anticoagulation with a VKA agent was previously indicated for mechanical mitral valve and permanent atrial fibrillation. The VKA used was acenocoumarol, as warfarin was unavailable in our country. The correction of coagulation disorders was obtained by the administration of fresh frozen plasma and by switching the anticoagulant agent to LMWH. Antibiotic therapy (carbapenem and vancomycin) most likely influenced the anticoagulant activity of acenocoumarol, and INR values increased outside of the therapeutic range resulting in nose bleedings. We did not discontinue anticoagulant treatment due to an increased cardioembolic risk determined by the presence of a metallic mitral valve, permanent atrial fibrillation, and a history of chronic microvascular cerebral ischemic disease. We switched the antibiotic agent, switched the VKA to LMWH, and administered fresh frozen plasma. All these interventions corrected the coagulation disorders and the clinical evolution.

## 4. Conclusions

DVIE is a complex disease with a challenging diagnosis. The evolution and treatment depend on etiology, the moment of onset related to valve implantation, age, and comorbidities. Clinical presentation can be specific, but in some etiologies, like EFIE, the clinical course may be subacute with nonspecific signs and symptoms. Although there is guidance for antibiotic treatment of EFIE in the guidelines, side effects and complications can develop, evolution may worsen, and antibiotic changes must be made. Anticoagulant treatment in IE is only necessary when there are associated indications, like atrial fibrillation or prosthetic valves, as in our patient. Otherwise, anticoagulation may interfere with antibiotic treatment and complicate the evolution. Surgery has precise indications, but it is not destitute of complications.

To the best of our knowledge, this case-based narrative review is the first report of a patient with simultaneously native and prosthetic double valve *Enterococcus faecalis* IE. Aside from the rare etiology of IE, another notable feature was the atypical clinical presentation with challenging therapeutic evolution that required a holistic approach without the need and indication for surgical intervention. Clinicians should be aware of the severity of DVIE in a patient with comorbidities and carefully monitor disease evolution, medication side effects, and surgical indications.

## Data Availability

Not applicable.
